# 

**Published:** 1881-10

**Authors:** 


PAGE

ORIGINAL COMMUNICATIONS—Have Birds the Faculty of Articulate Speech? By William A. Hammond, M.D. . . . . . 227
Veterinary Education. By Erskine S. Bates, M.D., D.V.S . . . . . 230
The expansion of the Horse's Foot when Shod and Unshod. By John N. Navin, V.S . . . . . 245
Chronic Endometritis in a Mare, with Enormous Distention of the Uterus. By L. E. Wheat, V.S., and W. H. Porter, M.D . . . . . 249
Pachy-meningitis Interna—Reticulated Round Cell Sarconia of Dura-Mater and Exostosis of the Inner Table of the Skull . . . . . 253

EDITORIAL—Bogus Veterinary Medical Degrees—New Veterinary Colleges—The Treasury Cattle Commission—The Appearance of Unknown Diseases – Pet Animals and Contagious Diseases—What Profession Shall a Young Man Choose?—Ensilage and Disease Again—Hydrophobia Among the Dogs in New York—National Cattle Commission—Vaccinating Animals—Veterinary Medical Register . . . . . 257–264

REVIEWS—A Treatise on the Diseases of the Ox—The Cat—Narvin's Veterinary Practice—The Management and Diseases of the Dog . . . . . 265

CASE DEPARTMENT—A Case of Rabies—The Epizooty at San Francisco—Spinal Paralysis in the Hyena and Puma—Contagious (?) Keratitis in the Cow—Ulceration of the Œsophagus in a Mule—A Three Days' Western Experience—Catarrhal Phthisis in a Cow—A Case of Suppurating Epididymitis—Urethral Calculus in a Bay Gelding . . . . . 267–279

PROGRESS OF VETERINARY SCIENCE—Fouquet's Method of Producing Either Sex—A New Parasite in Pork—Lampas—Malaria in Horses—Treatment of Chicken Cholera—Article on the Horse—A New Horse—Three Foals at a Birth—How to Kill Animals with a Blow—Intestinal Concretions in the Monkey—Splenic Fever in the Dog—The Dog and Tapeworm in Iceland—The Distance Animals Can Swim—Death from Rupture of the Rectum—How Ostriches Digest—The Blood in Hibernation—Upon the Presence of the Virus of Rabies in the Cerebral Substance of Mad Animals—The Turcoman Horse—String-Halt—Prevention and Treatment of Milk Fever—Pasteur's Experiment in Vaccinating Animals—Chicken Cholera—Liver Rot in Sheep . . . . . 280–288

NEWS AND MISCELLANY—New Veterinary Journal—Siberian Plague—Disease of the Eyes—Pimples, etc—New Veterinary Medical Colleges—Pen and Plough—Peculiar Colic—Anthrax in Louisiana—Alum for Cows in Summer—Item from Northern New York—Old and New English Racehorses—Female Boa Constrictor—Euphorbia Villosa—Death of a Famous Jersey Cow—Exportation of Contagious Pleuro-pneumonia to England—A Curious Epidemic—Canadian Cattle Healthy—New Veterinary Publications—A "Cure" for Hydrophobia—Gratitude—Dr. Noah Cressy—Mr. Graves on the Care of Snakes—Milk as a Cause of Death—The Manure Nuisance in New York—England and Pleuro-pneumonia in American Cattle—Monster Turtle—Another New Cattle Disease—An Alligator Sunstruck—Phagedæna: Is it a New Disease?—Food for Wild Beasts—Splenic Fever in Dutchess County, New York—The Horse and the Man—The Epizooty Again—Veterinary Education—The Horse Disease in St. Louis—A Veterinarian Selling Diseased Cattle—A Strange Disease—The Horse Fever—A Curious Deformity—Animal Pests in Greenwood Cemetery—A Good Move—Treatment for Scab—Evolution and Hybridism in Ornithology—"Firing" Horses—Tapeworm . . . . . 289–300

